# Structural connectivity at a national scale: Wildlife corridors in Tanzania

**DOI:** 10.1371/journal.pone.0187407

**Published:** 2017-11-02

**Authors:** Jason Riggio, Tim Caro

**Affiliations:** Department of Wildlife, Fish and Conservation Biology, University of California Davis, Davis, California, United States of America; Auburn University, UNITED STATES

## Abstract

Wildlife corridors can help maintain landscape connectivity but novel methods must be developed to assess regional structural connectivity quickly and cheaply so as to determine where expensive and time-consuming surveys of functional connectivity should occur. We use least-cost methods, the most accurate and up-to-date land conversion dataset for East Africa, and interview data on wildlife corridors, to develop a single, consistent methodology to systematically assess wildlife corridors at a national scale using Tanzania as a case study. Our research aimed to answer the following questions; (i) which corridors may still remain open (i.e. structurally connected) at a national scale, (ii) which have been potentially severed by anthropogenic land conversion (e.g., agriculture and settlements), (iii) where are other remaining potential wildlife corridors located, and (iv) which protected areas with lower forms of protection (e.g., Forest Reserves and Wildlife Management Areas) may act as stepping-stones linking more than one National Park and/or Game Reserve. We identify a total of 52 structural connections between protected areas that are potentially open to wildlife movement, and in so doing add 23 to those initially identified by other methods in Tanzanian Government reports. We find that the vast majority of corridors noted in earlier reports as “likely to be severed” have actually not been cut structurally (21 of 24). Nonetheless, nearly a sixth of all the wildlife corridors identified in Tanzania in 2009 have potentially been separated by land conversion, and a third now pass across lands likely to be converted to human use in the near future. Our study uncovers two reserves with lower forms of protection (Uvinza Forest Reserve in the west and Wami-Mbiki Wildlife Management Area in the east) that act as apparently crucial stepping-stones between National Parks and/or Game Reserves and therefore require far more serious conservation support. Methods used in this study are readily applicable to other nations lacking detailed data on wildlife movements and plagued by inaccurate land cover datasets. Our results are the first step in identifying wildlife corridors at a regional scale and provide a springboard for ground-based follow-up conservation.

## Introduction

Globally, land conversion and habitat degradation have resulted in many local wildlife extirpations and, as a consequence, populations are increasingly restricted to reserves isolated by agriculture and urbanization (e.g. [1–3]). Populations that lack connectivity to other protected areas can suffer from an inability to disperse between protected areas, compromised genetic variability within isolated populations due to lack of immigration, an inability of dwindling populations to be rescued from extirpation, and reduced opportunities for range shifts in response to global climate change [4–6]. Indeed some argue that the long-term viability of wildlife species relies on maintaining connectivity between protected areas (e.g. [6–8]), and it is widely acknowledged that large-scale conservation corridors might serve as such linkages between habitats (e.g. [9–14]).

There are many ways to identify wildlife corridors. One method is to ask reserve managers and wildlife researchers where corridors might exist. This can be an accurate and cost-effective method in places where people live or work, but may miss corridors where people simply are not looking. Another way would be to use GPS and/or VHF-collars to determine functional connectivity. This is often cost-prohibitive, however, and necessarily limited to local, or at best, regional scales. A third way to identify where wildlife corridors exist between protected areas is to model structural landscape connectivity using data derived from satellite imagery.

Landscape connectivity models combine environmental variables and their expected resistance to animal or plant movement as determined by expert opinion (and increasingly by actual animal movement data via GPS-collars) [15]. Inputs include land use data, which designate how people use land, and land cover datasets, which describe the physical features that cover the earth’s surface [16]. As anthropogenic land cover, such as cropland or urban extent, is generally considered a barrier to wildlife movement (e.g. [17–18]), precise identification of these regions is critical when creating accurate landscape connectivity models. Unfortunately, discriminating cropland from natural land cover using classification algorithms can be very difficult, particularly in savanna Africa: fallow crop fields are spectrally similar to neighboring dry grassland and shrubland/woodland without leaf cover during the dry season when cloud-free images are captured. As such, there is substantial disagreement over the extent of anthropogenic land cover among existing datasets on the continent [19–22].

Another issue with landscape connectivity models is lack of data validating their results. The best method for verifying the use of predicted corridors is through actual animal movement data gathered via VHF or GPS collars [15]. However these methods are time consuming, costly, and rely on frequent animal movement between habitat patches or on seasonal migratory patterns. Instead, interviews with people living within or adjacent to wildlife corridors may provide accurate information on wildlife movements that can be obtained relatively quickly and cheaply. These data can then be used to validate connectivity models (e.g. [23–24]).

Tanzania is an ideal country to test methods for delineating wildlife corridors, because it is the only country in Africa to have assessed its wildlife corridors at the national scale [25]. These assessments were published in a 2009 Tanzania Wildlife Research Institute (TAWIRI) publication [25] and a 2010 TAWIRI elephant management report [26], and identified a total of 34 corridors in Tanzania. These corridors were recognized as (A) unconfirmed corridors including both historical migration routes without recent confirmation and the shortest distances between protected areas without regard to land cover, (B) corridors of continuous natural lands between protected areas with no information on wildlife movements, (C) corridors of continuous or semi-continuous natural lands between protected areas with anecdotal information on wildlife movements, (D) routes of known animal movements between protected areas, or (E) formally proposed corridors linking habitats containing endangered species [27]. The authors of these reports subjectively classified the conservation urgency of each corridor, ranking their condition as *moderate*, *critical*, or *extreme* meaning that they likely had less than twenty, five, or two years, respectively, before closure to wildlife movement. In this way, more than two-thirds (24) of the corridors were assessed to be in *critical* or *extreme* condition, that is, likely to become closed within five years [26–27].

Though extremely useful, there are problems with such methods, however, because these corridor identification schemes are (i) an amalgamation of structural and functional elements, (ii) often rely only on the movement of one species, elephants (*Loxodonta africana*), (iii) rely extensively on researcher or hunter presence in an area, and (iv) frequently resort to anecdotal information. For example, of the 34 corridors noted by TAWIRI, the majority (22) lacked systematic animal movement data (types A, B, C or E), while six lacked land cover data (Type A) [26–27]. It would clearly be useful to develop alternative and more systematic methods for the identification of structural corridors and to assess their state of jeopardy with greater certainty.

To address these issues with corridor identification, the main goal of our research was to develop a single, consistent methodology to systematically assess wildlife corridors at a national scale using Tanzania as a case study. Specifically, our research aimed to answer the following questions; (i) which corridors may still remain open (i.e. structurally connected) at a national scale, (ii) which have been potentially severed by anthropogenic land conversion (e.g., agriculture and settlements), (iii) where are other remaining potential wildlife corridors located, and (iv) which protected areas with lower forms of protection (e.g., Forest Reserves and Wildlife Management Areas) may act as stepping-stones linking more than one National Park and/or Game Reserve. The first three questions aimed to assess the effects of anthropogenic disturbance on connectivity network configuration at a national scale. It was not our central goal to reassess wildlife corridors across Tanzania using the *ad hoc* approach and five methods as per Jones and colleagues [25], but rather to create a single quantitative assessment of all possible connections between protected areas in Tanzania and then to compare back to TAWIRI’s results and state whether or not the corridors they identified were structurally “open” or “severed” given our methodology. Given very little information on wildlife movement for most species in Africa (and the prohibitive cost of collecting it), our assessment focused on identifying structural connectivity based on landscape “naturalness” [28] between protected areas at the scale of large savanna mammals.

## Materials and methods

### Study area

Tanzania is a county of critical conservation importance containing 77 Important Bird Areas [29], 12 Centers of Plant Diversity [30], ten Global 200 Ecoregions [31], nine Endemic Bird Areas [29], eight Alliance for Zero Extinction Sites [32], five regions containing intact large mammal assemblages [33], two Biodiversity Hotspots [34–35], and one High-Biodiversity Wilderness Area [36]. The nation now has a stated commitment to maintaining connectivity between protected areas [37] and the 2009 Wildlife Act of Tanzania states “The Minister may, in consultation with relevant local authorities and by order in the *Gazette*, designate wildlife corridors, dispersal areas, buffer zones and migratory routes [38].” Nonetheless, rapid land use changes in Tanzania including agricultural expansion, dramatic deforestation for charcoal production, and road construction are threatening to sever [39–44] or have already closed [45–46] several corridors to wildlife movements.

Wami-Mbiki Wildlife Management Area in eastern Tanzania is a multi-use conservation area managed by the Wami-Mbiki Society (WMS) and composed of an approximately 2,500 km^2^ “core area,” and a 1,500 km^2^ “buffer zone” containing 24 member villages and farmland [47]. We chose to study wildlife corridors around Wami-Mbiki Wildlife Management Area for three reasons. First, Wami-Mbiki was highlighted in the original TAWIRI wildlife corridor report [25] as being linked to at least four surrounding protected areas, thus acting as a natural experiment to judge different conditions facing landscape connectivity in Tanzania. Second, the Wildlife Management Area and surrounding region represents a wide variety of environmental conditions as it sits at the confluence of three biomes (East African Coast, Somali-Maasai, and Zambezian). And third, Wami-Mbiki is surrounded by a heterogeneous mix of natural and converted lands and as such is likely to be representative of future conditions over the majority of Tanzania given projections about human population increase.

### Interviews

**Fig 1:**
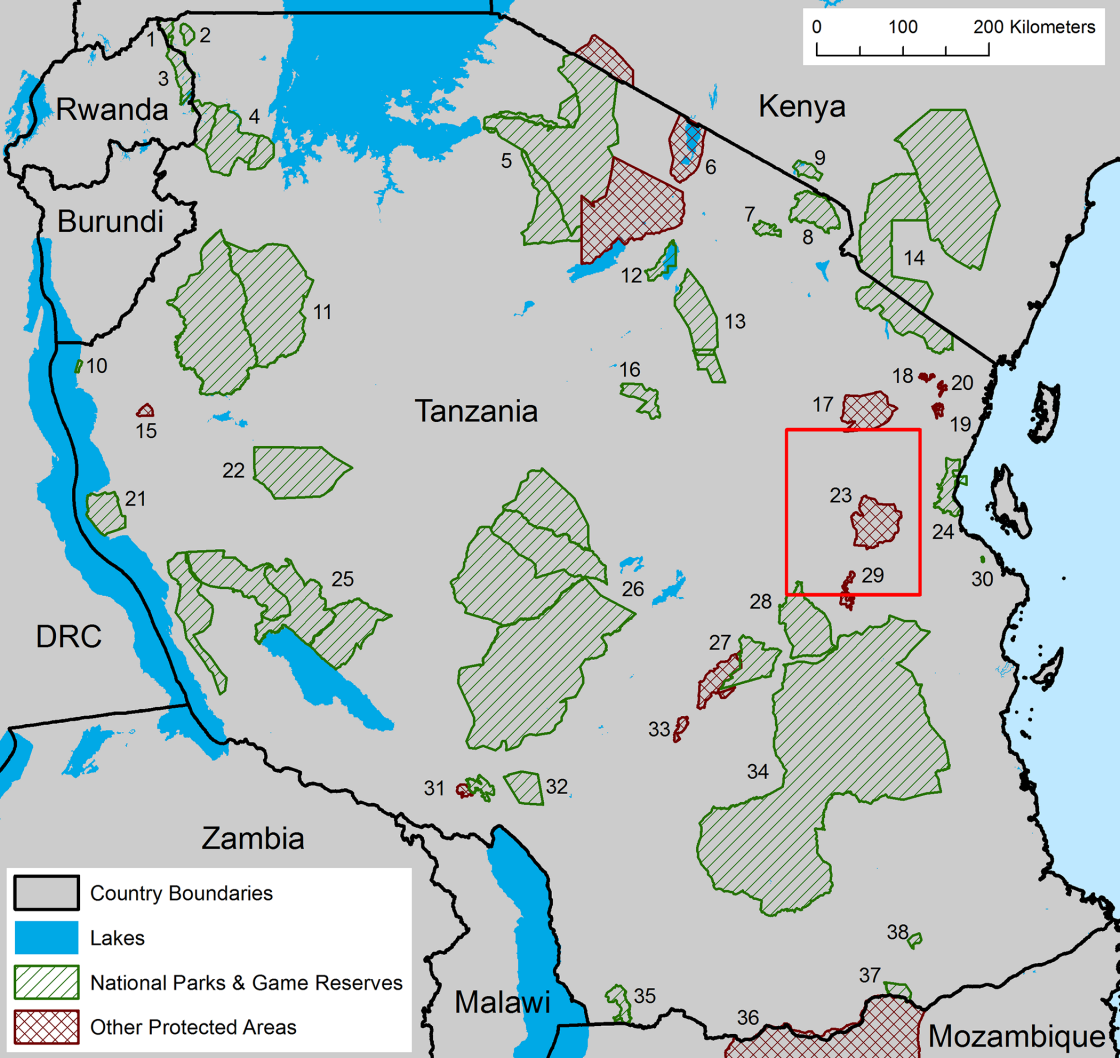
Map of protected areas and complexes linked by wildlife corridors in and adjacent to Tanzania. 1, Ibanda GR; 2, Rumanyika GR; 3, Akagera NP; 4, Burigi-Biharamulo Complex; 5, Serengeti-Ngorongoro Complex; 6, Lake Natron Basin; 7, Arusha NP; 8, Kilimanjaro NP; 9, Amboseli NP; 10, Gombe Stream NP; 11, Moyowosi-Kigosi Complex; 12, Lake Manyara NP; 13, Tarangire Complex; 14, Tsavo-Mkomazi Complex; 15, Uvinza FR; 16, Swaga Swaga GR; 17: Handeni GCA; 18, Baga and Kisima-Gonja FRs; 19, Amani NR; 20, Kambai FR; 21, Mahale Mountains NP; 22, Ugalla GR; 23, Wami-Mbiki WMA; 24, Saadani NP; 25, Katavi-Rukwa Complex; 26, Ruaha-Rungwa Complex; 27, Udzungwa Complex; 28, Mikumi NP; 29, Uluguru NR; 30, Pande GR; 31, Rungwe-Kitulo Complex; 32, Mpanga-Kipengere GR; 33, Uzungwa Scarp NR; 34, Selous GR; 35, Liparamba GR; 36, Niassa National Reserve; 37, Lukwika-Lumesure GR; and 38, Msanjesi GR. Red box shows the extent of the 80 villages with interview data concerning wildlife movement around Wami-Mbiki Wildlife Management Area.

In January and November 2014, JR and a Tanzanian translator conducted semi-structured interviews in Swahili concerning large mammal presence and the locations of wildlife corridors in 65 villages surrounding the Wildlife Management Area (Riggio et al. *in prep*). For each survey we first gained the permission of the Village Executive Officer and/or Village Chairperson to conduct interviews with members of the Village Natural Resource Committee or Village Game Scouts (mean number of participants = 3.8, range = 1 to 9). We introduced our research to the potential participants and gained their consent before proceeding. The Village Executive Office and/or Village Chairperson were usually present for and participating in the interview. We based our interview questions on a similar survey of villages concerning wildlife corridors in the Greater Wami-Mbiki Ecosystem by Van de Perre and colleagues [23]. Interview questions included “Do you think there is a path (corridor) that animals use to move from Wami-Mbiki?”, “Where is this path located (show on map)?”, “Where do the animals go?”, “Which species use this path?”, “What time of year do animals use this path?”, “Do the animals move across cultivated land?”, “How do you know about this path?”, and “Do you think this path will disappear? Why?”. If the response to the first question was no, we followed up with several questions including, “Was there a path used by animals?”, “Is there something blocking the path of animal movement?”, and “When did the path become blocked?”. We also included interview data from 15 villages between Wami-Mbiki and Saadani National Park (from [44]), therefore 80 villages in total (Fig 1). Fieldwork and interviews were conducted under the authority of the Tanzania Wildlife Research Institute, Wildlife Division, and the Commission for Science and Technology (COSTECH) (Research Permit # 2013-334-NA-2013-76).

### Wildlife corridor models

Using ESRI ArcGIS v.10 we created 12 cost surface layers of Tanzania using a 0.01° x 0.01° raster dataset (projected cell size of ~1km at the equator) of anthropogenic land conversion (GE Grids) [48]. The GE Grids dataset was created by visually evaluating high-resolution Google Earth imagery for the presence of anthropogenic land cover cell-by-cell across a raster overlaid on the imagery by the program (analyzed imagery dated between 2001 and 2016 with >90% from 2010 onwards). A cell was classified as “converted” if 50% or more of the land was converted to human land cover (e.g., croplands, built-up areas, and housing units such as bomas) [48]. We updated the GE Grids dataset where possible with the newest available imagery in regions where only 30 m resolution Landsat imagery had existed prior.

Three layers held cells containing natural lands at a cost of 1 to cross, while cells containing conversion were assigned values of 10, 100 or 1000. To create the remaining cost surface layers we ran each of those cost surfaces through the Focal Statistics tool using the “mean” metric with a 3 * 3, 5 * 5, 7 * 7, and 9 * 9 cell neighborhood. This tool assigns each cell the average value of the cells within the neighborhood around that target cell. The values of the cells in the resulting layers range from 1 to 10 (or 100 or 1000) depending on the cost surface input. A cell having a value of 1 is unconverted and surrounded only by unconverted cells in its neighborhood, while a cell having a value of 10 (or 100 or 1000) is converted and surrounded only by converted cells in its neighborhood. Values between 1 and 10 (or 100 or 1000) represent cells containing a varying amount of anthropogenic land conversion in its neighborhood. These 15 cost surface layers represent our uncertainty about both the magnitude and scale of the impact of anthropogenic land conversion on wildlife movement [49–50]. By selecting costs of 10, 100, or 1000 we are testing different assumptions of how difficult it is for an animal to move across converted cells, or the magnitude of impact. We address scale by comparing the impact of land conversion both on that cell alone (is wildlife movement only impacted through the converted cell itself?) and on the natural cells that surround the converted pixel (do converted cells influence the movement of wildlife through natural unconverted cells in their neighborhood?).

With these 15 cost surface layers as inputs, we modeled a subset of wildlife corridors in Tanzania using least-cost methods with Linkage Mapper [51–52] between Wami-Mbiki Wildlife Management Area in eastern Tanzania and all of its neighboring protected areas (clockwise from north to south: Handeni Game Controlled Area, Saadani National Park, Selous Game Reserve and Mikumi National Park) (Fig 1). For analyzing landscape connectivity we chose least-cost instead of circuit theory methods using Circuitscape [53–54] as circuit theory methods require long processing time at large scales [9], can be challenging to interpret [6], and lack a quantifiable and objective method for delineating distinct wildlife corridors [55]. As a single cell-wide least-cost path is unlikely to represent wildlife movement, we mapped least-cost corridors by selecting the lowest 5, 10, 15 and 20% cost cells in the resulting corridor map [56–57].

We compared the resulting 60 corridor model outputs (15 cost surfaces, mapped at 4 widths) to interview results on the location of wildlife movements around Wami-Mbiki. Locations of known wildlife movement are defined as a circle created from a 5 km buffer around the midpoint between two villages saying wildlife cross their village land to and/or from Wami-Mbiki Wildlife Management Area. Non-corridor locations are defined as circles created by a 5 km buffer around villages where interviewees reported no known wildlife movement. We used a 5 km buffer as this is the average distance between interviewed villages. We generated a confusion matrix and calculated Cohen’s kappa for each model based on which of the 75 locations the resulting pathways crossed. We considered the best model as the one having the highest kappa value of the 60 outputs while requiring the lowest number of cells to achieve agreement with the interview data. Taking into consideration that large inland water bodies (not present around Wami-Mbiki) pose a barrier to mammal movement, we masked out a combination of two raster water layers to each cost surface layer: classified lakes and reservoirs from the Global Lakes and Wetlands Database (GLWD-3) [58] and water bodies from the FAO Global Land Cover Database (GLC-SHARE layer 11) [59].

Using this model and method, we modeled wildlife corridors between all National Parks and Game Reserves in Tanzania using Linkage Mapper [52]. Corridors were dropped if they intersected another protected area. Protected areas were linked together to form complexes where reserves with the highest forms of protection (National Parks, National Reserves and Game Reserves) are contiguous (i.e. share a common border) (Fig 1). We also modeled corridors between all remaining areas noted in the TAWIRI reports [25, 26], which included corridors linking areas other than National Parks and Game Reserves (e.g., connections between Forest Reserves and protected areas in adjacent countries). Some wildlife corridors identified by TAWIRI were located between contiguous protected areas. Wildlife corridors cannot be modeled between habitat patches that lie next to each other, so instead we reviewed the high-resolution satellite imagery on Google Earth to determine if land conversion had divided these protected areas. If these corridors had not been divided they are labeled as contiguous.

We examined whether any open or potentially severed corridors crossed areas containing potential natural barriers (i.e. areas having slope >10° [42, 60] using a 30 m digital elevation model of the study area, or permanent wetlands [61]) based on the classified permanent wetlands from the Global Lakes and Wetlands Database (GLWD-3) [58]). To determine if wildlife corridors were likely to be severed by anthropogenic land conversion in the future we ran the unmodified GE Grids layer through the Focal Statistics tool, but this time used the “maximum” metric and a 3 * 3 neighborhood. The maximum statistic assigns each cell the maximum value of the cells surrounding it (0 for natural lands and 1 for converted), reclassifying any natural cell adjacent to a converted cell as converted. This method assumes that the natural lands closest to current croplands are the most likely to be converted in the future. While lands adjacent to converted lands might currently have some form of lesser protection (e.g., Forest Reserves, Wildlife Management Areas or Game Controlled Areas), it is a precautionary approach to assume that, despite current status, those lands are most likely to be converted next. Finally, we identified protected areas with lower forms of protection (e.g., Forest Reserves and Wildlife Management Areas) that lie within the identified corridors that may act as stepping-stones linking more than one protected area (National Park or Game Reserve).

## Results

### Interviews

The interview data confirmed the presence of wildlife movements at 34 locations, while an additional 41 locations contained no known wildlife movement. The cost surface layer with converted cells assigned a value of 10, with a 5 * 5 focal mean neighborhood, mapped at a width of the 15% lowest cost cells, resulted in the highest kappa value (0.68; S1 Fig). However, we chose the cost surface layer with converted cells assigned a value of 10, with a 3 * 3 focal mean neighborhood, mapped at a width of the 10% lowest cost cells as the best model, because it achieved nearly as high kappa (0.65) while requiring one-third fewer cells to capture the interview data (S1 Fig). Interviews conducted in 2014 indicated that farms and villages had severed the Wami-Mbiki to Handeni Game Controlled Area corridor identified by Jones and colleagues in 2009 [25]. Based on this information, we designated wildlife corridors as open if they had cumulative cost-weighted distance values less than the Wami-Mbiki to Handeni Game Controlled Area corridor (i.e. had lower resistance to cross; 145.2 weighted km). We classified corridors as potentially severed (i.e. likely closed to wildlife movement) if the linkage had a higher cumulative cost-weighted distance value than that threshold. The only exception to this rule was for the corridor linking the Udzungwa Complex to Selous Game Reserve. As modeled the corridor bypasses converted lands between the two protected areas, and follows natural lands to the north through Mikumi National Park. Thus the model predicts that the corridor as presented by TAWIRI [25] is potentially closed to large mammal movement. To avoid classifying corridors as potentially severed due to their length alone, we retained corridors with higher cumulative cost-weighted distance values if they had Euclidean distances greater than the Wami-Mbiki to Handeni Game Controlled Area corridor (81.2 km), and also do not cross converted lands (S1 Table).

### Wildlife corridors

**Fig 2:**
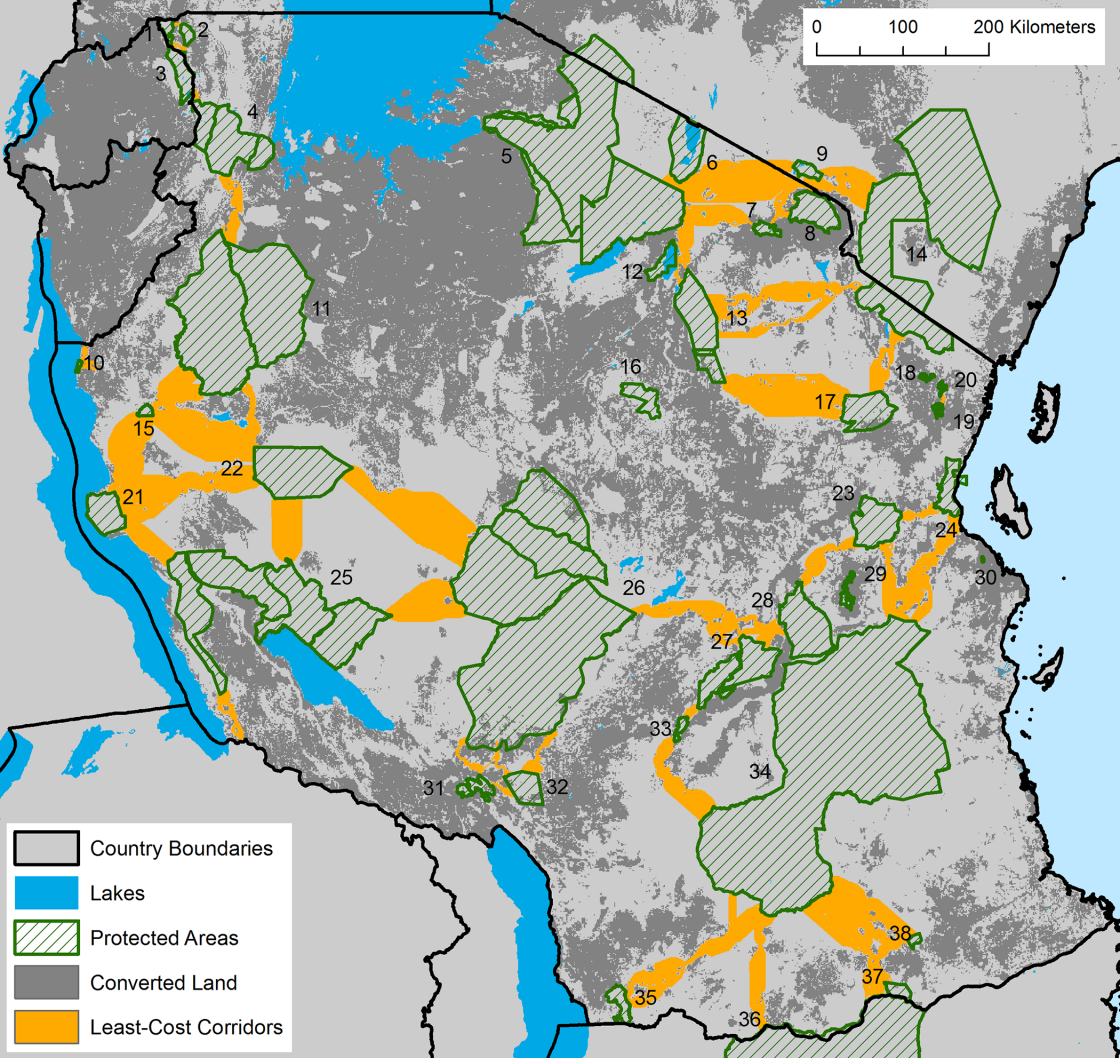
Locations of least-cost corridors linking protected areas in and adjacent to Tanzania. Least-cost corridors are shown representing the lowest 10% (orange) cumulative-cost cells. Numbers refer to protected area names listed in Fig 1 and S1 Table.

We identified 47 potential wildlife corridors open to wildlife movement; i.e. paths of continuous natural land cover between protected areas. Visual inspection of Google Earth imagery showed that all five of the contiguous corridors identified by TAWIRI were not severed by land conversion. This brings the total number of open wildlife corridors in Tanzania to 52 (47 and 5 contiguous corridors) (S1 Table; Fig 2). Many open corridors (N = 21, 40%) cross land likely to be converted in the future and are the most likely to be severed without timely conservation attention (S1 Table). Finally, our analysis shows that land conversion has potentially severed 23 wildlife corridors across Tanzania (S1 Table). So in total, we report 75 (52 open + 23 severed) potential wildlife corridors across Tanzania.

Of the 52 potential wildlife corridors identified as currently open by the least-cost corridor analysis, 23 were not actually discussed by the TAWIRI reports (S1 Table). Five corridors identified by TAWIRI are now classified as potentially severed (S1 Table). These were corridors between (i) Gombe Stream National Park and Masito-Ugalla Game Reserve (via Uvinza Forest Reserve), (ii) Ruaha-Rungwa Complex and Swaga Swaga Game Reserve, (iii) Tarangire Complex and Swaga Swaga Game Reserve, (iv) Udzungwa Complex and Selous Game Reserve, and (v) Wami-Mbiki Wildlife Management Area and Handeni Game Controlled Area. Twenty-one of the original 24 corridors that TAWIRI suggested might be severed by 2015 (*Critical* and *Extreme* urgency) are structurally connected, however (S1 Table).

**Fig 3:**
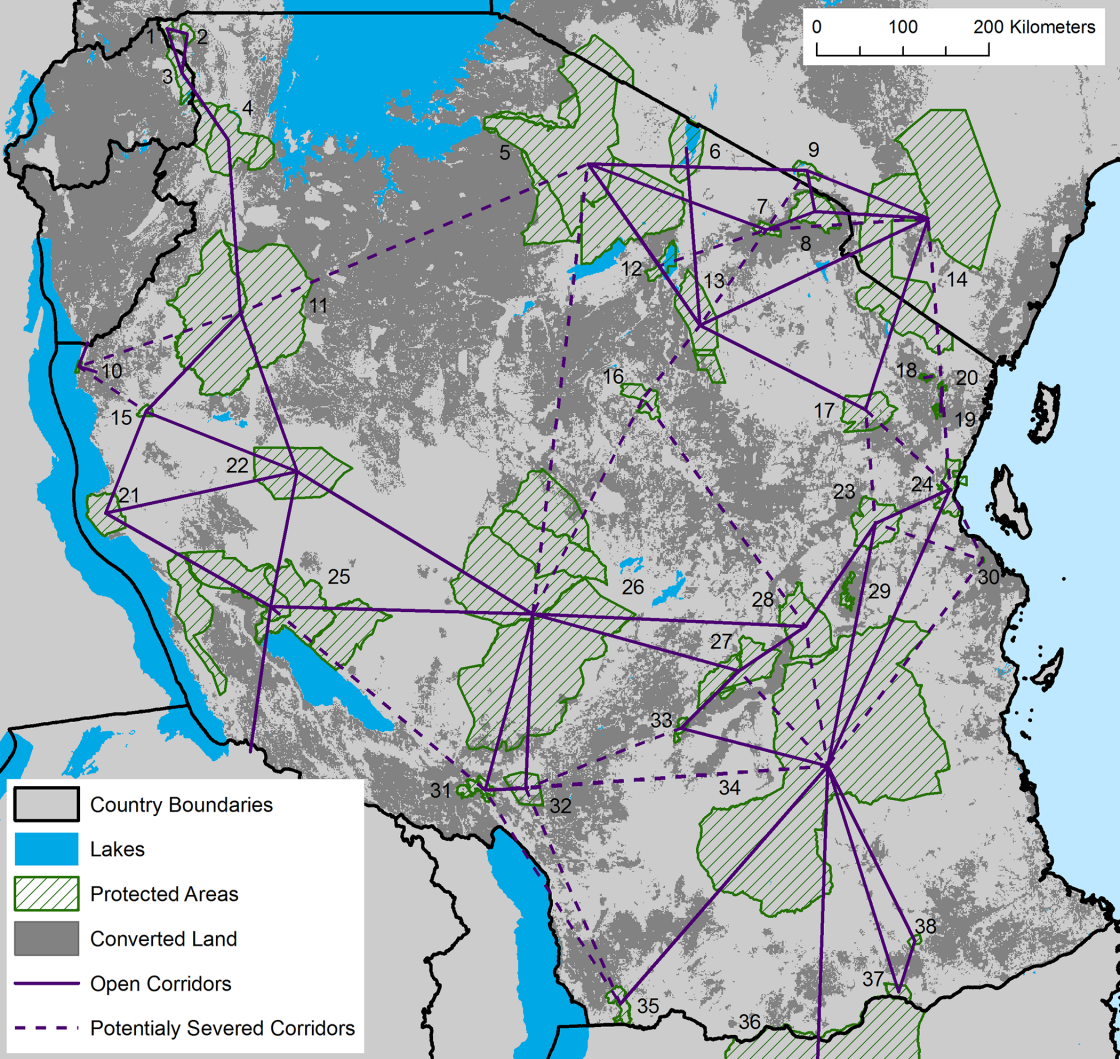
Network graph of open (solid lines) and severed (dashed lines) wildlife corridors linking protected areas in and adjacent to Tanzania. Numbers refer to protected area names listed in Fig 1 and S1 Table.

Encouragingly, structural wildlife corridors still connect protected areas from east to west across the nation linking all major miombo (deciduous savanna woodland) protected areas (Fig 3); all savanna protected areas are linked together in the north of the country. No open wildlife corridors remain to link protected areas between northern and southern Tanzania, however. Furthermore, two reserves (Gombe Stream National Park and Pande Game Reserve) are completely isolated from all others in the country (Fig 3). Our analysis highlighted two stepping-stones with low levels of protection (i.e. not National Parks, Nature Reserves or Game Reserves): (1) Uvinza Forest Reserve (Fig 1; #15), which connects Moyowosi-Kigosi Complex, Mahale Mountains NP and Ugalla GR, and (2) Wami-Mbiki Wildlife Management Area (Fig 1; #23), which connects Saadani and Mikumi National Parks and Selous Game Reserve.

## Discussion

The TAWIRI research principally used reports of reserve managers and wildlife researchers and literature to identify corridors, uncovering a total of 34 across Tanzania. Our more systematic analysis used a novel method of measuring landscape connectivity, combining a landscape resistance map derived from the GE Grids land conversion map [48], with interview data to verify the model. This approach identified an additional 41 potential corridors (34 + 41 = 75 corridors reported in total) (S1 Table; Fig 3). Our analysis surprisingly suggests that there are 21 potential wildlife corridors structurally open for wildlife movement *in addition* to those identified by TAWIRI with a total of 52 remaining potential wildlife corridors in Tanzania based on examining patterns of land conversion (S1 Table).

Additionally, we identified two stepping-stones between National Parks and/or Game Reserves that enjoy only low forms of protection in the western and eastern parts of the country–Uvinza Forest Reserve and Wami-Mbiki Wildlife Management Area. Both of these reserves might facilitate connectivity between three protected areas. These stepping-stone reserves are an important finding because it indicates that both of these areas require greater governmental and NGO conservation commitment. A third point to note is that corridors in northern (around Arusha, Lake Manyara and Tarangire National Parks) [40–43] and eastern Tanzania (surrounding Mikumi National Park and Wami-Mbiki Wildlife Management Area) [48] are being converted to agricultural land rapidly (S1 Table), suggesting that formally protected corridors in these areas need to be established quickly.

In 2009 and 2010, Tanzania Government reports [25, 26] suggested that six corridors would be severed in two years and 18 corridors would have disappeared in five years. In other words, they suggested that 71% of their 34 corridors would be closed to animal movement by 2016. Our least-cost corridor analysis based on data collected by satellite between 2001 and 2016 (>90% from 2010 onwards), a time window that spanned those reports, shows that only three of these 18 critical corridors have been severed by land conversion (plus two additional corridors whose conservation urgency was originally classified as *moderate*; S1 Table). More specifically, in western Tanzania the (i) Gombe Stream National Park to Masito-Ugalla GR (via Uvinza Forest Reserve) identified by TAWIRI has likely been severed by land conversion, while in central Tanzania, corridors linking (ii) Ruaha-Rungwa Complex and (iii) Tarangire Complex to Swaga Swaga Game Reserve have likely been closed to large mammal movement. Finally, in eastern Tanzania, anthropogenic land conversion has likely severed connections between (iv) Udzungwa Complex and Selous Game Reserve, and (v) Wami-Mbiki Wildlife Management Area and Handeni Game Controlled Area. In corroboration, we can find ground-based information noting that corridors (iv above) [45] and (v above) (*this study*) have been recently severed, while recent surveys suggest that chimpanzee (*Pan troglodytes*) movement is unlikely between Gombe Stream National Park and Uvinza Forest Reserve due to human presence and land conversion (A. Collins and A. Piel, *pers*. *comm*.).

Finally, while our analysis showed that there are large areas of land in western Tanzania through which large mammals may be able to move, we find that major wildlife movements elsewhere are more constrained. The four corridors that potentially linked large mammal populations in northern Tanzania to those in southern Tanzania (thereby potentially connecting large savannah mammal populations across the North-South gradient for the whole of Africa) have likely been severed by land conversion: (i) Ruaha-Rungwa Complex to Serengeti-Ngorongoro Complex (via the unprotected floodplains connecting Lake Eyasi, Lake Kitangiri and the Sibiti and Wembere Rivers), (ii) Ruaha-Rungwa Complex to Swaga Swaga Game Reserve, (iii) Tarangire Complex to Swaga Swaga Game Reserve, and (iv) Wami-Mbiki Wildlife Management Area to Handeni Game Controlled Area (S1 Table; Fig 3). On-the-ground-research is immediately necessary in these four regions to determine if these findings are accurate, and how these linkages might be restored. Encouragingly, research in Africa has shown that large mammals can reestablish movements between protected areas when barriers are removed [62]. Thus, it may not be too late to restore broken corridors in locations of vital conservation importance. Restoration will necessitate laws formulated by the Tanzanian government, but they have already established models of local community partnerships with the government and NGOs to conserve wildlife via coexistence and limited exploitation (models include Ngorongoro Conservation Area, Game Controlled Areas and Wildlife Management Areas) and discussion of these and other alternatives needs more active attention.

Our findings are optimistic: there might be a far greater potential for large mammals to move between protected areas than was previously supposed. Whether or not mammals use these corridors is of course an open question; research is required to document the movements of mammals along these potential corridors via interviews, ground surveys and GPS collaring of target species to determine their functional use. A key question that needs to be addressed in comparing corridor identification methods is whether large mammal species move through agricultural areas or, more specifically, which species traverse which sorts of converted land and over what distances. We suspect that Jones and colleagues [25] overlooked many of these corridors as two-thirds link protected areas with little or no researcher presence (e.g., the suite of Game Reserves in the northwestern corner of the country) (T. Caro, *pers*. *obs*.).

Furthermore, our analysis indicates that either 77% of these TAWIRI corridors were incorrectly classified as “probably less than 5 years remaining” [27], or that our cost-weighted threshold is too low and that wildlife movements are functionally blocked before land conversion severs structural linkages. While it is difficult to separate these possibilities, the fact that five out of the original six corridors denoted as being in *extreme* condition do not seem to be severed by land conversion, as well as 16 out of the original 18 categorized as *critical* suggests that TAWIRI’s *critical* and *extreme* categorizations may have been overly pessimistic. Nonetheless, the loss of one-sixth of Tanzania’s wildlife corridors identified in 2009 underscores the rapid erosion of connectivity, and those corridors identified in the TAWIRI reports need to be protected quickly.

Our research carries some caveats. First, it is important to note that natural barriers can block open corridors for certain species (S1 Table). For example, corridors linking the Burigi-Biharamulo Complex through Akagera National Park to Ibanda and Rumanyika Game Reserves in the northwest of Tanzania must cross the Kagera River and its extensive papyrus swamps to avoid converted lands. The effect of wetlands and rivers as a natural barrier is certainly species-specific (e.g., in the Okavango Delta of Botswana they act as barriers to movement for cheetah (*Acinonyx jubatus*), spotted hyena (*Crocuta crocuta*), and wild dog (*Lycaon pictus*), but not for lions (*Panthera leo*) [61]). Similarly, permanent wetlands and rivers exist along the structural corridors linking Ugalla Game Reserve to the Moyowosi-Kigosi Complex and the Ruaha-Rungwa Complex to Mpanga Kipengere Game Reserve. Mountain ranges may also act as barriers for certain species such as elephants that avoid steep slopes [60, 63]. This situation may pertain to northern Tanzania where a high escarpment and numerous isolated mountains bound grassland valleys. For instance, Morrison and Bolger [42] showed that a migratory wildebeest (*Connochaetes taurinus*) population moving between Tarangire and Lake Manyara National Parks and the Lake Natron Basin used low-lying bottlenecks between mountains with slopes >10°. Thus, our method for identifying wildlife corridors likely overestimates landscape connectivity for certain species if natural barriers exist.

Second, our method likely underestimates the impact of other forms of human disturbance that might decrease connectivity for certain species without the conversion of natural lands. For example, transportation infrastructure (e.g., roads and railways), noise, fences, hunting, and harassment may all act as effective barriers to movement but only for certain species (e.g. [61, 64–65]). Nonetheless research has shown that even paved roads are not complete barriers to some migratory large mammals in Tanzania (e.g., elephant [66], and wildebeest [42]), and therefore we omitted roads from the analysis as we did not want to bias our results towards species that were most sensitive to road presence.

Third, it is important to reiterate that corridors identified through land use conversion are structural in nature. We acknowledge that detailed visual examination of 0.01° * 0.01° grid squares using Google Earth imagery to separate converted from natural land does not directly bear on the issue of whether there is a functional corridor between neighboring protected areas. Next steps are to establish whether or not and which species cross converted land or tolerate human disturbance including noise, lights, and roads in moving through unconverted land. Moreover, some species are known to risk crossing cropland, such as elephants moving from Udzungwa to Selous (Southern Tanzania Elephant Project, *unpublished data*), so certain barriers are semi-permeable to movement [67]. Nonetheless, the GE Grids classification of “naturalness” allows us to provide a systematic assessment of the extent to which protected areas are still structurally connected within a nation, and thus provide important information for decision-makers.

Our method of modeling landscape connectivity using spatial data on anthropogenic land conversion, combined with interviews to validate these models is readily applicable to other regions. We show that a rapid assessment of structural connectivity between protected areas at a national scale is not only feasible, but can also provide an accurate first step in identifying wildlife corridors. These data can then be used to guide further research into where wildlife corridors likely persist, giving wildlife managers a means to home in on potential paths of wildlife movement. We recommend this method as a first step before deploying costly GPS collars or time-consuming surveys to determine where functional connectivity might occur.

## Supporting information

S1 Fig Least-cost corridor model performance. Plots comparing Cohen’s kappa to least-cost corridors delineated by selecting the lowest 5, 10, 15 and 20% cost cells for each corridor model. Models are based on cost surfaces (CS) with values ranging from 1 to 10, 100 or 1000. The base cost surface layers are modified using the focal mean (FM) metric with a 3x3, 5x5, 7x7 or 9x9 neighborhood.
(TIF)

S1 Table Potential wildlife corridors in Tanzania. Corridors are sorted by cost-weighted distance in ascending order. Numbers in parentheses refer to Fig 1. CWD:EuD is the ratio between cost-weighted distance and Euclidean distance. CWD:LCP is the ratio between cost-weighted distance and least-cost path distance. Corridors noted with an X are those that currently cross converted lands or are likely to cross future converted land. Natural Barrier refers to whether or not a corridor crosses a potential natural barrier (e.g., slope >10° or permanent wetlands). Any instance of “N/A” (not applicable) refers to contiguous corridors. *denotes TAWIRI [25, 26] does not mention these corridors. ^#^These two corridors noted by Jones and colleagues [25] were combined into one connection (#27) by Mduma and colleagues [26].
(DOCX)

S1 File Spatial data. This zip folder contains the file “Tanzania_Wildlife_Corridors.tif”. This raster dataset is the final predicted wildlife corridor map of Tanzania resulting from the analysis as described in the Methods. The folder also includes a shapefile (“Tanzania_Protected_Areas.shp”) that contains all of the protected areas mentioned in this analysis, numbered according to Fig 1.
(ZIP)
