# Supplementary figures and images for: Structural connectivity at a national scale: Wildlife corridors in Tanzania
Source: PLoS One. 2017 Nov 2;12(11):e0187407. doi: 10.1371/journal.pone.0187407 (PMC5667852; doi:10.1371/journal.pone.0187407)

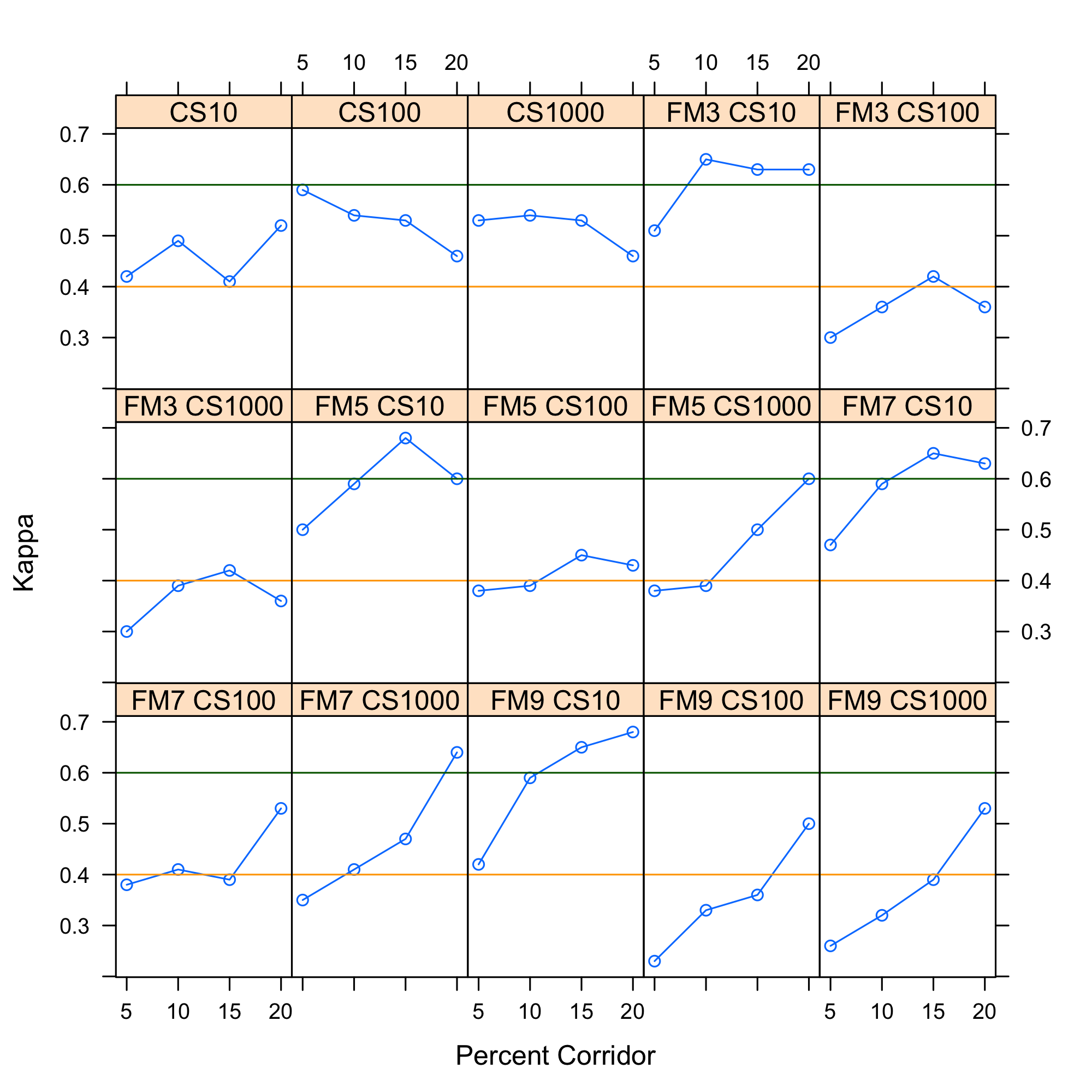

Supplement: S1 Fig — Plots comparing Cohen’s kappa to least-cost corridors delineated by selecting the lowest 5, 10, 15 and 20% cost cells for each corridor model. Models are based on cost surfaces (CS) with values ranging from 1 to 10, 100 or 1000. The base cost surface layers are modified using the focal mean (FM) metric with a 3x3, 5x5, 7x7 or 9x9 neighborhood. (TIF) [file pone.0187407.s001.tif]
