# Supplementary material for: Structural connectivity at a national scale: Wildlife corridors in Tanzania
Source: PLoS One. 2017 Nov 2;12(11):e0187407. doi: 10.1371/journal.pone.0187407 (PMC5667852; doi:10.1371/journal.pone.0187407)
Supplement: S1 Table — Corridors are sorted by cost-weighted distance in ascending order. Numbers in parentheses refer to Fig 1. CWD:EuD is the ratio between cost-weighted distance and Euclidean distance. CWD:LCP is the ratio between cost-weighted distance and least-cost path distance. Corridors noted with an X are those that currently cross converted lands or are likely to cross future converted land. Natural Barrier refers to whether or not a corridor crosses a potential natural barrier (e.g., slope >10° or permanent wetlands). Any instance of “N/A” (not applicable) refers to contiguous corridors. *denotes TAWIRI [25, 26] does not mention these corridors. #These two corridors noted by Jones and colleagues [25] were combined into one connection (#27) by Mduma and colleagues [26]. (DOCX) [file pone.0187407.s002.docx]

S1 Table: Potential wildlife corridors in Tanzania sorted by cost-weighted distance in ascending order. Numbers in parentheses refer to Fig 1. CWD:EuD is the ratio between cost-weighted distance and Euclidean distance. CWD:LCP is the ratio between cost-weighted distance and least-cost path distance. Corridors noted with an X are those that currently cross converted lands or are likely to cross future converted land. Natural Barrier refers to whether or not a corridor crosses a potential natural barrier (e.g., slope >10º or permanent wetlands). Any instance of “N/A” (not applicable) refers to contiguous corridors. *denotes TAWIRI [25, 26] does not mention these corridors. ^#^These two corridors noted by Jones and colleagues [25] were combined into one connection (#27) by Mduma and colleagues [26].

| **From** | **To** | **Euclidean Distance (EucD; km)** | **Cost-Weighted Distance (CWD; weighted km)** | **Least-Cost Path (LCP; km)** | **CWD:EucD** | **CWD:LCP** | **Least-Cost Corridor** | **Land Conversion** | **Future Conversion** | **Natural Barrier** | **Urgency (TAWIRI)** |
| --- | --- | --- | --- | --- | --- | --- | --- | --- | --- | --- | --- |
| Burigi-Biharamulo Complex (4) | Akagkera NP (Rwanda) (3) | 8.04 | 14.26 | 14.26 | 1.00 | 1.00 | Open |  |  | Wetland | Critical |
| Ibanda GR (1) | Burigi-Biharamulo Complex (4) (via Akagera NP - 3) | 9.15 | 15.37 | 15.37 | 1.68 | 1.00 | Open |  |  | Wetland | * |
| Udzungwa Complex (27) | Uzungwa Scarp NR (33) | 16.48 | 17.30 | 17.30 | 1.05 | 1.00 | Open |  |  | Slope | Critical |
| Baga FR (18) | Kisima-Gonja FR (18) | 0.58 | 17.54 | 2.68 | 30.04 | 6.54 | Open | X | X | Slope | Moderate |
| Kilimanjaro NP (8) | Amboseli NP (9) (Kenya) | 16.83 | 18.90 | 18.90 | 1.12 | 1.00 | Open |  |  |  | Critical |
| Ibanda GR (1) | Rumanyika GR (2) | 7.95 | 23.81 | 10.47 | 2.99 | 2.27 | Open | X | X | Slope | * |
| Gombe Stream NP (10) | Mukungu-Rukamabasi (Burundi) | 20.68 | 25.00 | 23.89 | 1.21 | 1.05 | Open |  |  | Slope | Critical |
| Rumanyika GR (2) | Burigi-Biharamulo Complex (4) (via Akagera NP - 3) | 16.64 | 30.65 | 24.54 | 1.84 | 1.25 | Open |  |  | Slope & Wetland | * |
| Kitulo-Rungwe Complex (31) | Mpanga-Kipengere GR (32) | 15.59 | 32.07 | 27.49 | 2.06 | 1.17 | Open |  | X | Slope | * |
| Udzungwa Complex (27) | Mikumi NP (28) | 11.26 | 33.17 | 30.26 | 2.95 | 1.10 | Open |  |  | Slope | Critical |
| Wami-Mbiki WMA (23) | Saadani NP (24) | 36.05 | 40.83 | 40.83 | 1.13 | 1.00 | Open |  |  |  | Extreme |
| Serengeti-Ngorongoro Complex (5) | Lake Manyara NP (12) | 14.91 | 47.77 | 47.77 | 3.20 | 1.00 | Open |  | X | Slope | Critical |
| Kambai FR (20) | Amani NR (19) | 8.50 | 61.06 | 15.18 | 7.18 | 4.02 | Open |  | X | Slope | Critical |
| Mahale Mountains NP (21) | Katavi-Rukwa Complex (25) | 62.39 | 64.65 | 64.65 | 1.04 | 1.00 | Open |  |  |  | Critical |
| Katavi-Rukwa Complex (25) | Zambia | 60.65 | 67.48 | 67.48 | 1.11 | 1.00 | Open |  |  |  | Extreme |
| Lake Manyara NP (12) | Tarangire Complex (13) | 22.73 | 73.43 | 44.06 | 3.23 | 1.67 | Open | X | X |  | Critical |
| Ugalla GR (22) | Katavi-Rukwa Complex (25) | 74.97 | 76.71 | 76.71 | 1.02 | 1.00 | Open |  |  |  | * |
| Msanjesi GR (38) | Lukwika-Lumesure GR (35) | 44.78 | 78.22 | 76.65 | 1.75 | 1.02 | Open |  |  |  | * |
| Kilimanjaro NP (8) | Tsavo-Mkomazi Complex (14) | 22.10 | 78.63 | 71.96 | 3.56 | 1.09 | Open |  |  |  | * |
| Katavi-Rukwa Complex (25) | Ruaha-Rungwa Complex (26) | 71.70 | 79.63 | 79.63 | 1.11 | 1.00 | Open |  |  |  | Moderate |
| Udzungwa Complex (27) | Selous GR (34) | 9.79 | 80.21 | 77.30 | 8.19 | 1.04 | Closed | X | X | Wetland | Critical |
| Moyowosi-Kigosi Complex (11) | Ugalla GR (22) | 70.29 | 80.48 | 80.48 | 1.14 | 1.00 | Open |  | X | Wetland | Not Assessed |
| Tsavo-Mkomazi Complex (14) | Handeni GCA (17) | 73.13 | 88.19 | 83.05 | 1.21 | 1.06 | Open |  | X |  | * |
| Mikumi NP (28) | Wami-Mbiki WMA (23) | 73.25 | 88.73 | 86.27 | 1.21 | 1.03 | Open |  | X |  | Extreme |
| Arusha NP (7) | Kilimanjaro NP (8) | 15.28 | 95.64 | 28.52 | 6.26 | 3.35 | Open | X | X |  | * |
| Ruaha-Rungwa Complex (26) | Mpanga-Kipengere GR (32) | 33.38 | 97.10 | 55.75 | 2.91 | 1.74 | Open | X | X | Wetland | Critical |
| Burigi-Biharamulo Complex (4) | Moyowosi-Kigosi Complex (11) | 67.56 | 100.53 | 91.10 | 1.49 | 1.10 | Open |  | X | Slope | Critical |
| Serengeti-Ngorongoro Complex (5) | Tarangire Complex (13) | 53.58 | 100.88 | 71.50 | 1.88 | 1.41 | Open | X | X | Slope | * |
| Tarangire Complex (13) | Lake Natron Basin (6) | 62.51 | 110.31 | 80.94 | 1.76 | 1.36 | Open | X | X |  | Extreme |
| Selous GR (34) | Msanjesi GR (38) | 103.00 | 110.47 | 110.47 | 1.07 | 1.00 | Open |  |  |  | * |
| Wami-Mbiki WMA (23) | Selous GR (34) | 85.26 | 110.68 | 94.01 | 1.30 | 1.18 | Open |  | X |  | Critical |
| Gombe Stream NP (10) | Kwitanga Forest (not shown) | 16.35 | 116.63 | 22.31 | 7.13 | 5.23 | Open | X | X | Slope | Critical |
| Ruaha-Rungwa Complex (26) | Rungwe-Kitulo Complex (31) | 37.58 | 121.88 | 62.00 | 3.24 | 1.97 | Open | X | X | Slope | * |
| Serengeti-Ngorongoro Complex (5) | Arusha NP (7) | 76.64 | 126.36 | 83.81 | 1.65 | 1.51 | Open | X | X | Slope | * |
| Selous GR (34) | Lukwika-Lumesure GR (37) | 124.83 | 133.41 | 133.41 | 1.07 | 1.00 | Open |  |  |  | Not Assessed |
| Udzungwa Complex (27) | Selous GR (34) (via Uzungwa Scarp NR - 33) | 105.98 | 137.55 | 134.63 | 1.30 | 1.02 | Open |  |  | Slope | * |
| Tarangire Complex (13) | Handeni GCA (17) | 130.67 | 139.67 | 139.67 | 1.07 | 1.00 | Open |  |  |  | * |
| Ruaha-Rungwa Complex (26) | Udzungwa Complex (27) | 106.35 | 142.86 | 140.18 | 1.34 | 1.02 | Open |  |  | Slope | Extreme |
| Selous GR (34) | Niassa National Reserve (36) (Mozambique) | 131.05 | 145.02 | 145.02 | 1.11 | 1.00 | Open |  |  |  | Moderate |
| Wami-Mbiki WMA (23) | Handeni GCA (17) | 81.21 | 145.20 | 101.84 | 1.79 | 1.43 | Closed | X | X |  | Critical |
| Lake Manyara NP (12) | Arusha NP (7) | 87.00 | 148.07 | 105.52 | 1.70 | 1.40 | Closed | X | X |  | * |
| Saadani NP (24) | Selous GR (34) | 136.36 | 157.98 | 153.26 | 1.16 | 1.03 | Open |  | X |  | * |
| Mahale Mountains NP (21) | Ugalla GR (22) | 147.00 | 158.00 | 158.00 | 1.07 | 1.00 | Open |  |  | Slope | * |
| Saadani NP (24) | Pande GR (30) | 57.44 | 164.26 | 74.34 | 2.86 | 2.21 | Closed | X | X |  | * |
| Arusha NP (7) | Tsavo-Mkomazi Complex (14) | 85.24 | 166.59 | 143.24 | 1.95 | 1.16 | Closed | X | X |  | * |
| Ugalla GR (22) | Ruaha-Rungwa Complex (26) | 156.22 | 168.30 | 168.30 | 1.08 | 1.00 | Open |  |  |  | * |
| Moyowosi-Kigosi Complex (11) | Mahale Mountains NP (21) (via Uvinza FR - 15) | 153.31 | 168.32 | 168.32 | 1.10 | 1.00 | Open |  |  | Slope | * |
| Ruaha-Rungwa Complex (26) | Mikumi NP (28) | 155.25 | 182.50 | 182.50 | 1.18 | 1.00 | Open |  | X | Slope | * |
| Tarangire Complex (13) | Tsavo-Mkomazi Complex (14) | 152.90 | 187.25 | 174.10 | 1.22 | 1.08 | Open |  | X |  | * |
| Mahale Mountains NP (21) | Ugalla GR (22) (via Uvinza FR - 15) | 171.83 | 189.41 | 189.41 | 1.10 | 1.00 | Open |  |  | Slope | Not Assessed |
| Selous GR (34) | Liparamba GR (35) | 156.58 | 192.57 | 192.57 | 1.23 | 1.00 | Open |  |  |  | * |
| Tarangire Complex (13) | Arusha NP (7) | 86.36 | 192.70 | 120.78 | 2.23 | 1.60 | Closed | X | X |  | * |
| Serengeti-Ngorongoro Complex (5) | Tsavo-Mkomazi Complex (14) (via Amboseli NP - 9) | 179.61 | 196.31 | 196.30 | 1.09 | 1.00 | Open |  |  | Slope | * |
| Saadani NP (24) | Handeni GCA (17) | 80.15 | 203.13 | 145.24 | 2.53 | 1.40 | Closed | X | X |  | * |
| Saadani NP (24) | Tsavo-Mkomazi Complex (14) | 129.59 | 210.61 | 153.06 | 1.63 | 1.38 | Closed | X | X |  | * |
| Wami-Mbiki WMA (23) | Pande GR (30) | 95.29 | 214.84 | 120.43 | 2.25 | 1.78 | Closed | X | X |  | * |
| Tarangire Complex (13) | Swaga Swaga GR (16) | 52.10 | 240.80 | 76.19 | 4.62 | 3.16 | Closed | X | X | Slope | Extreme |
| Selous GR (34) | Pande GR (34) | 102.98 | 243.53 | 159.17 | 2.36 | 1.53 | Closed | X | X |  | * |
| Ruaha-Rungwa Complex (26) | Swaga Swaga GR (16) | 128.10 | 259.01 | 212.42 | 2.02 | 1.22 | Closed | X | X |  | Moderate |
| Katavi-Rukwa Complex (25) | Kitulo-Rungwe Complex (31) | 193.29 | 281.21 | 220.22 | 1.45 | 1.28 | Closed | X | X | Slope | * |
| Gombe Stream NP (10) | Moyowosi-Kigosi Complex (11) | 91.48 | 310.41 | 145.81 | 3.39 | 2.13 | Closed | X | X | Slope | * |
| Kitulo-Rungwe Complex (26) | Selous GR (34) | 224.81 | 322.78 | 291.90 | 1.44 | 1.11 | Closed | X | X | Slope | * |
| Mpanga-Kipengere GR (32) | Selous GR (34) | 170.36 | 342.70 | 294.62 | 2.01 | 1.16 | Closed | X | X | Slope | * |
| Gombe Stream NP (10) | Masito-Ugalla GR (22) (via Uvinza FR - 15) | 197.68 | 375.29 | 230.78 | 1.90 | 1.63 | Closed | X | X | Slope | Moderate |
| Mpanga-Kipengere GR (32) | Udzungwa Complex (27) (via Uzungwa Scarp NR - 33) | 169.56 | 443.47 | 259.21 | 2.62 | 1.71 | Closed | X | X | Slope | * |
| Ruaha-Rungwa Complex (26) | Serengeti-Ngorongoro Complex (5) | 260.34 | 445.71 | 339.34 | 1.71 | 1.31 | Closed | X | X | Wetland | * |
| Kitulo-Rungwe Complex (26) | Liparamba GR (35) | 264.10 | 518.52 | 349.53 | 1.96 | 1.48 | Closed | X | X | Slope | * |
| Mpanga-Kipengere GR (32) | Liparamba GR (35) | 235.06 | 538.44 | 352.25 | 2.29 | 1.53 | Closed | X | X | Slope | * |
| Mikumi NP (28) | Swaga Swaga GR (16) | 252.63 | 612.44 | 482.55 | 2.42 | 1.27 | Closed | X | X | Slope | * |
| Moyowosi-Kigosi Complex (11) | Serengeti-Ngorongoro Complex (5) | 232.10 | 762.65 | 650.07 | 3.29 | 1.17 | Closed | X | X |  | * |
| Kitulo Plateau NP (31) | Rungwe NR (31) | N/A | N/A | N/A | N/A | N/A | Contiguous | N/A | N/A | N/A | Critical |
| Uluguru N (Uluguru NR - 29) | Uluguru S (Uluguru NR - 29) | N/A | N/A | N/A | N/A | N/A | Contiguous | N/A | N/A | N/A | Critical |
| Tarangire Complex (13) | Simanjiro GCA (not shown) | N/A | N/A | N/A | N/A | N/A | Contiguous | N/A | N/A | N/A | Critical |
| ^#^Loazi FR (not shown) | Kalambo FR (not shown) | N/A | N/A | N/A | N/A | N/A | Contiguous | N/A | N/A | N/A | Extreme |
| ^#^Katavi-Rukwa Complex (25) | Loazi FR (not shown) | N/A | N/A | N/A | N/A | N/A | Contiguous | N/A | N/A | N/A | Moderate |
| Tarangire Complex (13) | Lolisale GCA (not shown) | N/A | N/A | N/A | N/A | N/A | Contiguous | N/A | N/A | N/A | Moderate |
| Tarangire Complex (13) | Mkungunero GR (not shown) | N/A | N/A | N/A | N/A | N/A | Contiguous | N/A | N/A | N/A | Moderate |
